# Bears are simply voles writ large: social structure determines the mechanisms of intrinsic population regulation in mammals

**DOI:** 10.1007/s00442-014-2892-z

**Published:** 2014-01-31

**Authors:** Morten Odden, Rolf A. Ims, Ole Gunnar Støen, Jon E. Swenson, Harry P. Andreassen

**Affiliations:** 1 Faculty of Applied Ecology and Agricultural Sciences, Hedmark University College, Evenstad, 2480 Koppang, Norway; 2 Department of Arctic and Marine Ecology, University of Tromsø, 9037 Tromsø, Norway; 3 Department of Ecology and Natural Resource Management, Norwegian University of Life Sciences, 1432 Ås, Norway; 4 Norwegian Institute for Nature Research, 7485 Trondheim, Norway

**Keywords:** Dispersal, Infanticide, Intrinsic population regulation, Reproductive suppression, *Microtus*

## Abstract

The literature reveals opposing views regarding the importance of intrinsic population regulation in mammals. Different models have been proposed; adding importance to contrasting life histories, body sizes and social interactions. Here we evaluate current theory based on results from two Scandinavian projects studying two ecologically different mammal species with contrasting body sizes and life history traits: the root vole *Microtus oeconomus* and the brown bear *Ursus arctos*. We emphasize four inter-linked behavioral aspects—territoriality, dispersal, social inhibition of breeding, and infanticide—that together form a density-dependent syndrome with potentially regulatory effects on population growth. We show that the two species are similar in all four behaviors and thus the overall regulatory syndrome. Females form matrilineal assemblages, female natal dispersal is negatively density dependent and breeding is suppressed in philopatric young females. In both species, male turnover due to extrinsic mortality agents cause infanticide with negative effects on population growth. The sex-biased and density-dependent dispersal patterns promote the formation of matrilineal clusters which, in turn, leads to reproductive suppression with potentially regulatory effects. Hence, we show that intrinsic population regulation interacting with extrinsic mortality agents may occur irrespective of taxon, life history and body size. Our review stresses the significance of a mechanistic approach to understanding population ecology. We also show that experimental model populations are useful to elucidate natural populations of other species with similar social systems. In particular, such experiments should be combined with methodical innovations that may unravel the effects of cryptic intrinsic mechanisms such as infanticide.

## Introduction

“Although the devil is in the details of population control, and there is much biological interest in the quirks shown by many species… it is important for the management of our natural resources that useful generalizations are carefully analyzed and presented” (Krebs 2009).

The concept of population regulation implies negative density-dependent population growth (Turchin 1995). Accordingly, “intrinsic population regulation” occurs when animals lower their population growth rate through behavioral, physiological and genetic changes as a response to increasing population densities (Sinclair 1989; Krebs 2002). For conservation biology it is important to unravel the specific mechanisms of population regulation and their strength to mitigate populations from going extinct.

The literature reveals opposing views regarding the occurrence and significance of intrinsic regulation in mammals. In the paper entitled “Are big mammals simply small mammals writ large”, Caughley and Krebs (1983) claimed that there was a body-size difference in the mode of population regulation. Studies on small mammals often reported population regulation to be determined by intrinsic factors, whereas there was more support for extrinsic population regulation [predation, food, or diseases (see e.g., Stenseth and Ims 1993; Krebs 1996; Sinclair 2003)] in larger mammals (mammals with an intrinsic rate of increase <0.45 per year, and a body mass >30 kg). At that time, the hypothesis about size-dependent population regulation was based partly on group selection, as Caughley and Krebs (1983) argued that small mammals should be regulated by intrinsic factors to avoid extinction, due to their high rate of increase (but see Krebs 2009).

The size-dependent population regulation proposed by Caughley and Krebs (1983) was rejected by Erb et al. (2001), who used autoregressive models on population time series data to show that populations of both small and large mammals exhibited delayed density-dependent feedback likely to be due to extrinsic population-regulating factors.

Here, we do not advocate one hypothesis over the other. Rather we use data from a large (bear) and a small (vole) mammal to describe how intrinsic factors may contribute to the regulation of both small and large mammal populations. To some extent we therefore follow an idea of Wolff (1997), who argued that variation in population regulation was not between large and small mammals, but was rather determined by the species’ social structure. Wolff (1997) argued that intrinsic regulation would occur in populations in which female territoriality limited offspring rearing space, leading to positively density-dependent reproductive suppression. Moreover, he hypothesized that territoriality was adapted to avoid infanticide of altricial young committed by females (see also Wolff 1993). Intrinsic regulation was assumed to occur under a limited set of conditions, and therefore intrinsic regulation should be relatively uncommon compared with extrinsic regulation.

Wolff (1997) and Krebs (2009) described the following intrinsic density-dependent processes that may result in population regulation:

1. Territoriality, which limits breeding space as a population increases.
2. Positive density-dependent dispersal, which functions to regulate population dynamics (Stenseth 1983).
3. Reproductive suppression, which regulates population growth, as sexual maturation of weaned females and/or males is inhibited by the presence of dominant adult individuals.
4. Infanticide, which has a direct numeric effect on population size, as adult individuals kill neonates to improve their own reproductive success.

Territoriality seems to be a prerequisite for intrinsic population regulation, as it is expected to affect the other three mechanisms.

Here we examine whether the intrinsic mechanisms (territoriality, infanticide, dispersal, and social inhibition of breeding) highlighted by Krebs (2009) and Wolff (1997) occur in a small (the root vole *Microtus oeconomus*) and a large (the brown bear *Ursus arctos*) mammal, both with altricial young. We have studied these mechanisms intensively in two Scandinavian projects comprising a set of experimental studies of root vole populations and a long-term field study of brown bears. Actually, there are few cases found in the literature that could have been used in the same comparison, because all these mechanisms have seldom been studied in the same species. However, to some extent we also refer to studies of other species whenever they provide information relevant for our synthesis.

## Study species

The root vole and the brown bear have almost identical circumpolar distributions in the Northern Hemisphere. The body mass of sexually mature root voles in our experiments averaged 50 g. Adult female brown bears have a mean weight of 96 kg and adult males 201 kg in the spring, with fall weights 35–65 % higher (Swenson et al. 2007). Hence, root voles and brown bears may be used as representatives for small and large terrestrial mammals, respectively. Both species show substantial male-biased sexual size dimorphism. Adult male root voles of the two geographical races used in the experimental study are ca. 30–40 % heavier than females (Bondrup-Nielsen and Ims 1990), and adult male brown bears are twice as heavy as adult females.

This difference in body mass is reflected in the sizes of their activity areas. Root voles in our experiments utilized an average area of 400 m^2^ (range 60–4,000 m^2^), estimated by radio tracking (Ims et al. 1993; Andreassen et al. 1998), with males having three times larger home ranges than females [see also Lambin et al. (1992) for a similar result based on a study of a natural root vole population]. Female brown bear home ranges in southern Sweden were on average 217 km^2^ (range 81–999 km^2^), with males using on average 1,055 km^2^ (range 314–8,264 km^2^), thus having five times larger home ranges than females (Dahle and Swenson 2003a). In addition to these huge size differences, the two species also show different winter strategies. Brown bears hibernate, and root voles live actively under the snow cover.

With regard to habitat and diet, the root vole is a habitat specialist inhabiting swampy river bank vegetation (Tast 1966) and having a high proportion of graminoids and herbs in their diet (Soininen et al. 2009). In contrast, the brown bear utilizes a large array of habitats, mainly forested areas, but avoids habitats characterized by high human activity (Nellemann et al. 2007). They are omnivorous, with a varied diet that typically changes seasonally between carnivory and herbivory (Dahle et al. 1998).

As is common in small mammals, the root vole shows a typical* r*-strategy, with a high reproductive output and short life span. Females may mate when 3 weeks old, and give birth to a litter consisting of from two to 11 offspring every 3 weeks, as they may mate again the same day that they give birth. Voles that start to reproduce during the reproductive summer season seldom survive the winter (Aars and Ims 2002). In contrast, the brown bear shows a typical* K*-strategy, with low reproductive output and long life expectancy. In southern Sweden, the average age of female primiparity is 4.5 years, and the age of the first successful litter averages 5.2 years (Swenson et al. 2001). The mean litter size is 2.3 cubs and the litter interval is 1.6 years, but the interval between successfully weaned litters averages 2.4 years (Swenson et al. 2001).

These different life history strategies provide the potential for differences in population dynamics. The voles exhibit multiannual population cycles typical for small rodents in the Northern Hemisphere (Steen et al. 1990; Steen 1995; Kendall et al. 1998; Henden et al. 2011), where both extrinsic and intrinsic factors are likely to shape the population dynamics (Stenseth et al. 1996; Andreassen et al. 2013). Peak-year densities may become very high, with several hundred animals per hectare in optimal habitats (Aars and Ims 2002). The brown bear population today is regulated by wildlife management, but naturally has much more dampened population dynamics. The estimated number of brown bears in Sweden was 3,400 animals in 2008, with an annual population increase of 4.5 % (Kindberg et al. 2011). Over some large areas, the density can average ca. 30 bears per 1,000 km^2^ (Solberg et al. 2006), but locally, densities can exceed 60 bears per 1,000 km^2^ (Zedrosser et al. 2013). There is no population estimate for Norway, but a minimum of 166 individual bears was confirmed genetically to have been in Norway in 2010 (Tobiassen et al. 2011).

In spite of these contrasting characteristics, the two species actually show very similar spatio-social behavior. Individuals are primarily solitary in both species with some degree of intrasexual territoriality and a polygyneous mating system, which is quite common for several small rodent and large carnivore species (Le Galliard et al. 2012; Steyaert et al. 2012).

## Research projects

### The vole experimental system at Evenstad, Norway

The aim of the vole project, which started in 1989, has been to study the mechanisms driving the dynamics of small rodent populations, using the root vole as a model species. Root voles are good candidates for this kind of experimental model system (Ims and Stenseth 1989) due to their small size, short generation time, and small spatial requirements. The root vole has proved in many ways to be a very useful organism to elucidate population ecological questions. As a habitat specialist (Tast 1966), it is easy to assess and manipulate the habitat. Entire populations may be confined within relatively small areas, and their high affinity to live traps makes it easy to monitor population development and the characteristics of individuals during the snow-free season. Root voles carry radio-collars without any known side-effects (Johannesen et al. 1997), so that individual space use can be studied precisely (see e.g., Hansteen et al. 1997). The root vole is very easily held and bred in the laboratory, which give the opportunity for more controlled studies of life history traits (Ims 1994, 1997; dos Santos et al. 1995), as well as standardized experimental populations to be used in field experiments.

We have used a mechanistic approach in our studies, all of which were conducted in the framework of experimental model systems (EMS) (Ims and Stenseth 1989; Ims et al. 1993; Wiens et al. 1993). EMS are aimed at exploring empirical hypotheses at relatively small spatial and temporal scales and the experiments presented here were all conducted in enclosed plots. However, the fencing and the spatial scaling of the study plots in our experiments appeared to have little bearing on individual space use and reproductive tactics as the experimental voles appear to have the same basic characteristics in open populations (Tast 1966; Lambin et al. 1992; Gliwicz 2007). Also the spatio-temporal dynamics at the scale of local habitat patches or local demes appear to be little affected by the scale of the experimental population or meta-population (Glorvigen et al. 2013a, b). As usual in scientific experiments, we have attempted to control for factors that are not the focus. Hence, the experiments have all been initiated by releasing laboratory-raised animals, with known life history, into enclosed areas empty of mammalian predators and intra- and interspecific competitors other than the ones released. Each of the experiments has been run for several months (sometimes for up to 1 year) so that several generations of field-born animals have been included in the analyses. Matrilineal relatedness between individuals has been established by various methods (Aars et al. 1994; Andreassen and Ims 1998). Space use has been established using radio-telemetry (Andreassen et al. 1998; Andreassen and Ims 1998) and demographic parameters (including dispersal) by means of intensive live-trapping (Aars et al. 1999; Andreassen and Ims 2001). The habitat in the various plots consisted of dense meadow vegetation and has been maintained to be optimal for root voles. The actual habitat configuration employed in the different experiments appears to have had little bearing on how the experimental populations are intrinsically regulated (Ims and Andreassen 2000; Glorvigen et al. 2013b).

### The Scandinavian brown bear research project

The Scandinavian brown bear research project (SBBRP) started in 1984. The project has had two study areas: one in Northern Sweden and one in central Sweden and southeastern Norway. The southern study area consists of 13,000 km^2^ of intensively managed boreal forest in a rolling landscape sparsely populated by humans. The elevations range from about 200 to 1,000 m, but only a small part of the area is above the timberline (750 m). Bears are hunted intensively in the entire area. The northern study area encompasses 8,000 km^2^ and contains three mountainous national parks and adjacent forested land with low human influence. The area is characterized by deep valleys, glaciers, and high plateaus ranging up to 2,000 m. Bears are protected in the national parks, but hunted in the surrounding forested land. The forests in both study areas are dominated by Scots pine (*Pinus sylvestris*) and Norway spruce (*Picea abies*), but deciduous trees are common. The density of bears is lower and the home ranges are generally larger in the north (Dahle and Swenson 2003a).

The primary goals and ambitions of the SBBRP have been to: (1) document the basic ecology of the Scandinavian brown bear, (2) provide the management authorities with data and interpretations, and (3) provide information about brown bears to the general public. The primary method in the SBBRP is to follow individual female bears and their female offspring from birth to death, thus creating pedigrees, many of which now cover five generations or more. Male bears have also been monitored, but to a lesser extent. Bears are captured from helicopters and equipped with radio collars for monitoring (Arnemo et al. 2006). Only very high frequency collars were used until 2003, but lately Global Positioning System technology has been employed. From 1984 to 2012 a total of 482 individual bears have been radio-collared and monitored until death, where possible. An additional 340 bears have been captured and marked, but not radio-collared. Genetic analysis of bear tissues, hair, and scats has been an important tool in determining relatedness between radio-collared, hunter-killed and other dead bears from within and outside the study areas, and for population and density estimates. Basic and applied research have complimented each other in the project and have covered an array of research questions from genetics, social organization, mating system, life history, population ecology, behavior, predator–prey relations, and conflicts with humans.

## Comparison of intrinsic mechanisms

### Territoriality

Adult root voles are solitary, with larger home ranges and core areas in adult males than adult females (Andreassen et al. 1998)—a result that appears to be consistent among studies of small rodents in general (Ims 1987a, Le Galliard et al. 2012). Their degree of space sharing depends on sex and kinship (Bjørnstad et al. 1998; Andreassen et al. 1998; Andreassen and Ims 1998; Le Galliard et al. 2006). Adult males defend territories against other males, but their ranges overlap those of adult females (Rosell et al. 2008). Females’ home ranges overlap to a greater extent than among males, and their spatial overlap is positively associated with genetic relatedness (Andreassen et al. 1998; Andreassen and Ims 1998; Bjørnstad et al. 1998). Such a matrilineal population structure appears to be a basic trait in many species of arvicoline rodents (Johannesen et al. 2000, Le Galliard et al. 2012) as in most other mammal taxa (Armitage 1987).

As in root voles, adult brown bears are solitary with larger home ranges and core areas among males than females (Dahle and Swenson 2003a). Male home ranges overlap quite extensively, and to a greater extent than in adult females (Dahle and Swenson 2003a, b). Although neither of the sexes is considered strictly territorial in Scandinavia, some form of spatial dominance seems to occur, as both sexes show an inverse relationship between home range size and density that is not linked to food availability (Dahle and Swenson 2003a). The degree of home range overlap among females is positively associated with genetic relatedness (Støen et al. 2005). Moreover, similar to that found for root voles (Bjørnstad et al. 1998), the genetic relatedness in the population in brown bears declines significantly with geographic distance among females, but not among males or among individuals of the opposite sex (Støen et al. 2005).

Hence, the most prominent similarity in space use of root voles and brown bears is the influence of genetic relatedness in the female associations. Both species form aggregations of related individuals. These female kin groups, i.e., matriarchies, have been shown to function as social entities that inhibit immigration by unrelated individuals (Gundersen et al. 2001; Andreassen and Ims 2001; Andreassen et al. 2002; Støen et al. 2005), i.e., according to the social fence hypothesis (Hestbeck 1982). Male space use differs between the two species, as male root voles tend to be territorial (Fauske et al. 1997), whereas brown bear males are not (Dahle and Swenson 2003a, b).

### Natal dispersal

Dispersal is commonly divided into three phases: emigration, transition, and immigration (Ims and Yoccoz 1997; Andreassen et al. 2002). Root voles exhibit the basic characteristics of sex-specific natal dispersal common to many species of small mammals (Ims and Hjermann 2001; Lambin et al. 2004; Le Galliard et al. 2012). During natal dispersal, emigration rates are male biased (Gundersen and Andreassen 1998; Andreassen and Ims 2001). Males disperse longer distances than females, and a higher proportion of males are in the transition phase, i.e., males are more commonly unsettled and move into hostile habitats (Gundersen and Andreassen 1998; Aars et al. 1999). Emigration rates are inversely density dependent; individuals in habitat patches with the highest population density exhibit the lowest probability of emigrating (Andreassen and Ims 2001). However, those that disperse tend to move to patches of lower population density. Hence, most shifts occur from relatively low to even lower density patches (Andreassen and Ims 2001). This pattern of density dependence is most prominent in females, as males tend to disperse and settle independently of patch-specific densities (Aars and Ims 2000). The different dispersal strategies of males and females sometimes result in spatially uneven operational sex ratios especially some distance away from large source populations (Aars and Ims 2000). Although the main aspects of condition-dependent dispersal in root voles appear to be largely similar to those documented for many other small mammal species [i.e., inversely density-dependent female dispersal rate (Le Galliard et al. 2012)], the experimental studies on root voles are exceptional in terms of their ability to dissect mechanisms operating in different stages of the dispersal process (e.g., Andreassen and Ims 2001) as well as documenting the regulating effect of dispersal on population dynamics (e.g., Ims and Andreassen 2000, 2005).

Among brown bears, subadult males emigrate at higher rates and move longer distances once they disperse than subadult females (Støen et al. 2006a; Zedrosser et al. 2007). Density has a negative effect on both the probability of dispersal and dispersal distances, and stronger inverse density dependence is more evident among females than among males (Støen et al. 2006a; Zedrosser et al. 2007). As a consequence, the density of females declines rapidly from core areas, and males dominate in the periphery (Swenson et al. 1998). Dispersing females are less associated with their mother in the period between weaning and dispersal than philopatric sisters (Zedrosser et al. 2007). This and the fact that philopatric females have higher body masses indicate competition for philopatry among females (Støen et al. 2006a; Zedrosser et al. 2007).

In both root voles and brown bears, males disperse farther than females, leading to more young males in the periphery of an expanding population. Furthermore, the inverse density-dependent female dispersal in both species implies that social fences (see above) inhibit dispersal in dense populations, a mechanism promoting the formation of matrilineal clusters.

### Social inhibition of breeding

In root voles, reproductive suppression occurs through mother-daughter dominance relationships (Gundersen and Andreassen 1998; Ims and Andreassen 2000; Le Galliard et al. 2007). Sexual maturation is often delayed among philopatric young females (Gundersen and Andreassen 1998; Le Galliard et al. 2007)—again a trait that appears to be pervasive among arvicoline rodent species (Le Galliard et al. 2012). Also in brown bears, philopatric daughters exhibit a significantly delayed primiparity (5.2 years) as compared with dispersing daughters (4.3 years) (Støen et al. 2006b). Neighboring females tend to breed asynchronously (Ordiz et al. 2008), a pattern suggesting that social inhibition of breeding is a significant factor affecting their reproductive success.

Among young female root voles and brown bears, a decision to stay in the natal range implies a cost in terms of reproduction, and the strategy of staying is more common when population densities are high (Andreassen and Ims 2001; Støen et al. 2006a).

### Infanticide

Experiments conducted to simulate turnover of male root voles, i.e., predation and subsequent immigration of unfamiliar individuals, show that this disruption of the social system significantly lowers population growth due to a lower survival of adult females and lower number of recruits (Andreassen and Gundersen 2006). The lower adult survival was unexpected and difficult to explain. However, an inverse relationship between survival and the degree of space sharing among females in treatment plots suggests an influence of intrasexual interactions. The lower recruitment was partially due to delayed reproduction, but more importantly, was due to a lower survival of nestlings. Although causes of death were not observed directly, the lower survival was assumed to be caused by infanticidal immigrant males. Infanticide apparently has evolved three times in rodents, in Sciuromorpha, Myomorpha, and Caviomorpha, but there is no evidence that it is sexually selected in these groups (Blumstein 2000). The development of new methods to more directly observe and document the frequency of infanticide in the field is much needed in case of small mammals (Opperbeck et al. 2012).

Swenson et al. (1997, 2001) recorded survival of the cubs of radio-collared female brown bears in two areas: one with a high level of human hunting of male bears, and thus high adult male turnover and subsequent immigration; and another area with little adult hunting and therefore lower adult turnover. Cub survival was significantly lower in the area with hunting of adult males, and the effect lasted for ~1.5 years following the killing of an adult male. In brown bears, all requirements for infanticide to be sexually selected are fulfilled; it shortens the time to the mother’s next estrus (Steyaert et al. 2013b), infants are not killed by their father, and the perpetrators sire the next litter (Bellemain et al. 2006). Infanticide occurs most commonly in litters of primiparous females (Zedrosser et al. 2009). Furthermore, females with cubs-of-the-year restrict their range size and have lower quality diets than other bears in the mating season and increase their ranges and have diets of similar quality to other bears in the post-mating season, thus suggesting a strategy to avoid contact with infanticidal males that has a nutritive cost (Dahle and Swenson 2003b; Steyaert et al. 2013a, c). Apparent sexually selected infanticide is common in fissiped carnivores with relatively long lactation periods relative to gestation periods (van Schaik 2000).

Although infanticide appears to occur in both study species, we have not observed female bears committing infanticide and mortality of root vole nestlings is not dependent on the presence of unrelated females. It has, however, been observed sporadically in studies of the brown bear in other parts of the world, although adult males dominate among the known perpetrators (Hessing and Aumiller 1994; McLellan 1994).

## Discussion

Caughley and Krebs (1983) recognized that their view of size-dependent population regulation was influenced by different methodological approaches to studies of behavior and population dynamics in small and large mammals. Effects of sociality on population dynamics are difficult to detect in nature, especially among species that are large, long-lived, rare, or for other reasons difficult to observe. Hence, current information on the demographic effects of sociality stem mainly from experimental studies of smaller and less space-demanding species that are amendable to manipulative treatments, as we have shown here for the root vole. The idea that larger mammals rarely exhibit self-regulation may therefore be due to the difficulties in obtaining relevant data. The Scandinavian brown bear project is one of the few large mammal studies from which these kinds of data actually have been obtained.

In contrast to Erb et al. (2001), Caughley and Krebs (1983) and Krebs (2009) limited their review of regulatory mechanisms in mammals to include only herbivores. Interspecific size differences in herbivores are strongly linked to differences in both taxonomic relatedness and social structure. Larger mammalian herbivores are commonly ungulates, whose social structure rarely is based on territoriality. A large proportion of studies on smaller herbivorous mammals have been on small rodents, which in most cases exhibit some form of territoriality. Hence, it is difficult to disentangle the relative importance of body size (and intrinsic rate of increase) and social structure in models of intrinsic population regulation in herbivorous mammals.

In 2009, Krebs (2009) revised his hypothesis about population regulation in mammals. He argued that the role of intrinsic and extrinsic factors in regulating small mammal populations is still controversial and supported Wolff’s (1997) predictions that intrinsic population control is most probable in small mammals with altricial young. Krebs (2002) also requested further studies on population regulation in predatory mammals and more frequent experimental studies on the mechanisms of population regulation.

Here we have followed the requests of Krebs (2002, 2009) and taken a mechanistic approach with detailed long-term observational studies on a large carnivore species (brown bear) and a series of experimental studies on a small herbivore (root vole), both of which have been effective in terms of unraveling intrinsic mechanisms of population regulation. Our comparison of these two species with quite contrasting life history traits confirmed that both brown bears and root voles are affected by all four intrinsic factors suggested by Wolff (1997) and Krebs (2009); i.e., territoriality, infanticide, dispersal and social inhibition of breeding. However, the effects of these four intrinsic factors do not follow Wolff’s (1997) model.

Wolff (1997) predicted that intrinsic regulation in mammals occurred mainly in populations where offspring-rearing space was limited by female territoriality, which ultimately was induced by the risk of infanticide by other females (see also Wolff 1993). The root vole and the brown bear did not meet these conditions. First, root vole nestlings and brown bear cubs faced risks of infanticide, but from males, not from females (Swenson et al. 1997; Andreassen and Gundersen 2006). Second, females were not strictly territorial, but seemed rather to have a flexible territorial system with a higher degree of overlap among kin (Bjørnstad et al. 1998; Fauske et al. 1997; Andreassen et al. 1998; Andreassen and Ims 1998; Støen et al. 2005). Hence, territoriality may be best described as a continuous scale between an ideal despotic and an ideal free distribution of reproducing individuals (Fretwell and Lucas 1970). Potential regulatory effects due to territoriality should thus vary accordingly.

Patterns of dispersal and reproductive suppression in root voles and brown bears do indicate that offspring-rearing space occasionally may be limited. Young female root voles and brown bears chose philopatry instead of dispersal as a response to high densities of conspecifics (Andreassen and Ims 2001; Støen et al. 2006a; Zedrosser et al. 2007), despite the fact that this often delayed their breeding due to reproductive suppression (Gundersen and Andreassen 1998; Støen et al. 2006b; Le Galliard et al. 2007). Presumably the chance of dispersing and reproducing successfully is sufficiently low at high population densities to outweigh the reproductive cost of philopatry. Negative density-dependent dispersal indicates that some form of spatial dominance exists, and that “social fences” may contribute to regulate population dynamics (Hestbeck 1982; Gundersen et al. 2002). Our studies propose that social fences are products of kin groups, matriarchies, where daughters are reproductively inhibited by their mothers.

As Wolff (1997) predicted, dispersal was negatively density dependent in both root voles and brown bears (Andreassen and Ims 2001; Støen et al. 2006a; Zedrosser et al. 2007). Hence, dispersal in itself will not be a population-regulating factor (see also Ims and Andreassen 2005). However, negative density-dependent dispersal, in combination with reproductive suppression, will function as a mechanism to regulate populations through the creation of matriarchies and social inhibition of breeding in young females (see also Wolff 1997; Krebs 2009).

Wolff (1997) acknowledged that his model would seldom be realized, due to the combination of several necessary criteria. We have shown that infanticide committed by males has strong population consequences and may actually result in a decrease in root vole populations in the middle of the breeding season (Andreassen and Gundersen 2006). However, infanticide is probably not at all common in undisturbed natural populations. In both brown bears (Swenson et al. 1997, 2001; Zedrosser et al. 2009) and root voles (Andreassen and Gundersen 2006) the contribution of infanticide to population performance resulted from a disruption of the social system due to the extrinsically induced turnover of adult, dominant males, even though infanticide is sexually selected in bears and probably not in voles (van Schaik 2000). For voles such turnover is mostly due to pulses of high predation rates (Ims and Andreassen 2000), while for bears it is mostly due to hunting by humans (Zedrosser et al. 2013). Hence, infanticide is not a constant pressure for individual females as this intrinsic factor interacts with extrinsic determinants of male mortality. Infanticide may be a contributing factor for the evolution of territoriality, but our studies do not give any support to the hypothesis that territoriality is an adaptation to avoid female infanticide, in contrast to the large array of studies showing the significance of food resources in shaping female sociality (e.g., Ostfeld 1985, 1990; Ims 1987a, b).

In conclusion, we have shown that inter-linked intrinsic mechanisms together form a density-dependent syndrome that is likely to contribute to the population regulation of both a small herbivore and a large carnivore. Our results are surprising, as these two species have contrasting life history strategies. Obviously, it is the similarities in behavior that yield similar intrinsic mechanisms in both species. Hence, we reject Caughley and Krebs’ (1983) hypothesis that body size and intrinsic rate of increase are determinants of population regulation mechanisms. We also reject Wolff’s (1997) model of population regulation, as female infanticide does not seem to be very common and therefore not an evolutionary force of female territoriality. Some kind of female territoriality is probably a crucial component of intrinsic population regulation. However, territoriality seems to be a rather flexible behavior. The presence of kin groups (or demes) may be a better entity to describe territoriality, as they may create exclusivity of space along with other social aspects through social fences.

The similar way intrinsic factors affect root vole and brown bear populations indicates that these behaviors associated with sociality and dispersal have been exposed to similar and strong evolutionary forces. However we acknowledge that we have not shown how important intrinsic factors are relative to extrinsic factors. Clearly the susceptibility of voles and bears to extrinsic factors is very different. Root voles live often under a massive predation pressure (Steen 1995; Ims and Andreassen 2000), possibly acting in delayed density-dependent manner so as to give rise to extreme multi-annual population cycles (Stenseth et al. 1996). Root vole population dynamics are also liable to be strongly affected by stochastic adverse weather episodes during the winter (Aars and Ims 2002; Korslund and Steen 2006). Recent studies have shown that, when subjected to extreme weather events in the high Arctic, populations of large herbivores may be affected in an astonishingly similar way to populations of *Microtus* voles (Stien et al. 2012, Hansen et al. 2013). For Scandinavian bears, however, neither natural enemies nor weather effects are important, and human actions serve as the main extrinsic impact on the population. For both species, however, we emphasize that extrinsically induced mortality interacts with intrinsic mechanisms of the population (cf. Stenseth et al. 1996; Andreassen et al. 2013), in particular dispersal and infanticide, so as to shape population demography. Indeed, our review of the many recent studies conducted on the two species shows the significance of similar mechanistic approaches to achieve a better understanding of population ecology. We agree with Krebs (2002) that it is not enough to search for density-dependent patterns by statistical time-series analyses, but that we need to understand which factors affect population growth rates in order to support decision makers and managers. The synthesis we have provided here also serves as a justification of small-scale empirical experimental models for generalizing population mechanisms. We envisage further development of such experimental models, in particular by combining them with new methods for unraveling cryptic mechanisms of intrinsic population regulation such as infanticide.
